# A Text Messaging–Based Program to Transition From Basal Insulin to Glucagon-Like Peptide-1 Receptor Agonists in Safety-Net Diabetes Care: Pilot Quality Improvement Intervention Study

**DOI:** 10.2196/76993

**Published:** 2026-04-09

**Authors:** Natalie Levy, Katie Nerlino, Sherlane Bongalos, Alex Dasilva, Chinye Uzor, Ying Jie Liang, Olubunmi Sonubi

**Affiliations:** 1 Division of General Internal Medicine and Clinical Innovation, NYU Grossman School of Medicine, OBV - A620, 462 First Avenue, New York, NY, 10016, United States; 2 Department of Medicine, NYC Health + Hospital/Bellevue, New York, NY, United States; 3 Department of Nursing, NYC Health + Hospitals/Bellevue, New York, NY, United States

**Keywords:** type 2 diabetes, basal insulin, basal insulin dosing, glucagon-like peptide-1 receptor agonists, GLP-1 receptor agonists, glucagon-like peptide-1 receptor agonist dosing, GLP-1 receptor agonist dosing, primary health care, safety net, delivery of health care, SMS text messaging, telemedicine, mobile health, mHealth, eHealth, health care disparities, social drivers of health

## Abstract

**Background:**

Glucagon-like peptide-1 receptor agonists (GLP-1 RAs) and basal insulin both lower blood sugar, but while insulin puts people at risk of hypoglycemia and weight gain, GLP-1 RAs do not. In addition, GLP-1 RAs have added cardiometabolic and renal benefits. For these reasons, when possible, many primary care providers prefer their patients with *well-controlled* type 2 diabetes to be *transitioned* from basal insulin to a GLP-1 RA. This transition process can be labor intensive, requiring multiple dosing adjustments and a watchful eye for hypoglycemia and hyperglycemia. The Mobile Insulin Titration Intervention (MITI)–GLP1 program uses SMS text messaging–based technology to support a streamlined and supervised transition process from basal insulin to a GLP-1 RA. This program takes place at a multilingual safety-net clinic.

**Objective:**

Our objectives were to assess program feasibility and acceptability to determine whether the intervention was doable, practical, and worthy of further investigation via a larger controlled trial. Preliminary clinical outcomes are also discussed in this paper.

**Methods:**

Patients were enrolled on a secure web platform that sent them a daily SMS text message asking the following: “What was your fasting blood sugar this morning?” Each weekday, texted responses containing patients’ fasting blood sugar levels were checked for alarm values, and once weekly, patients were called and advised on whether and how to lower their basal insulin and increase their GLP-1 RA dose. The program was co-run by general internal medicine physicians and nurses and continued until the patient had their insulin stopped completely and/or their GLP-1 RA dose reached the maximum, or 16 weeks elapsed. All enrolled patients were included in the analyses.

**Results:**

A total of 72 patients completed the pilot program. Feasibility and acceptability were high. Of 3671 SMS text messages sent by the program, 3520 (95.89%) received a response from patients. Of 719 cumulative weeks in which Thursday titration phone calls were attempted, successful connections with patients were made in 649 (90.26%) instances. Preliminary clinical outcomes were promising. Insulin doses were meaningfully reduced (55/72, 76.39% had their basal insulin reduced by at least 50%; 45/72, 62.5% had their insulin stopped completely). GLP-1 RA doses were meaningfully increased (64/72, 88.89% had their GLP-1 RA dose increased by ≥1 level; 45/72, 62.5% were discharged on the maximum dose of their GLP-1 RA). There was minimal hypoglycemia (5/3520, 0.14% of the SMS text messages reported a value of <80 mg/dL) and hyperglycemia (1/3520, 0.03% of the SMS text messages reported a value of >400 mg/dL).

**Conclusions:**

A general internal medicine–run MITI-GLP1 pilot program using SMS text messaging and interdisciplinary teamwork between internists and nurses is a feasible and acceptable intervention for safely and effectively transitioning people with well-controlled type 2 diabetes away from basal insulin and toward a GLP-1 RA.

## Introduction

Insulin lowers blood sugar but introduces the risk of hypoglycemia and weight gain [1-5]. Glucagon-like peptide-1 receptor agonists (GLP-1 RAs) also lower blood sugar. However, unlike insulin, GLP-1 RAs are associated with a low risk of hypoglycemia and support weight loss [6,7]. In addition, studies have shown that GLP-1 RAs improve meaningful clinical outcomes for many people with type 2 diabetes (T2D), including reducing cardiovascular events in adults with T2D and established or high risk of atherosclerotic cardiovascular disease, reducing heart failure–related symptoms and physical limitations in adults with T2D and symptomatic heart failure with preserved ejection fraction and obesity, and slowing down the progression of chronic kidney disease in adults with T2D and chronic kidney disease [8-17]. Thus, many general internal medicine physicians would prefer their patients to be maximized on GLP-1 RAs with their insulin minimized.

Adding a GLP-1 RA to the regimen of a patient who is already on basal insulin *and* who has *well-controlled* blood sugar requires careful adjustment and monitoring [18,19]. If the basal insulin dose is lowered too quickly, blood sugar can rise, and the patient is at risk of “losing faith” in the new GLP-1 RA medication. (They may request to return to the basal insulin that previously kept their blood sugar well controlled.) On the other hand, if the basal insulin is not lowered quickly enough in a patient who has a robust blood sugar–lowering response to the GLP-1 RA (eg, because of increased pancreatic production of insulin or due to side effects that lead to decreased oral intake), hypoglycemia can occur. Frequent check-ins with successive small titrations allow the transition to proceed without leading to blood glucose values that are too low or too high. They also ensure that any large deviations from what had been the baseline, well-controlled blood sugar level are noticed immediately.

The Mobile Insulin Titration Intervention (MITI)–GLP1 is a program that supports the transition from basal insulin to GLP-1 RAs in people with well-controlled T2D. It was designed and carried out in a primary care internal medicine safety-net clinic in New York City, New York, where many patients face barriers related to social drivers of health. To the best of our knowledge, it is the only study in the literature to examine the use of an SMS text messaging–based program to adjust 2 different classes of diabetes medication simultaneously: basal insulin and GLP-1 RAs. Our objectives in this study were to assess program feasibility and acceptability to determine whether the intervention was doable, practical, and worthy of further investigation via a larger controlled trial. Preliminary clinical outcomes such as the decrease in insulin, increase in GLP-1 RAs, and avoidance of hypoglycemia and hyperglycemia during the transition are also discussed in this paper.

## Methods

### Overview

Bellevue Hospital is a safety-net hospital and part of NYC Health + Hospitals, the largest public health care system in the United States. Patients at the Bellevue Hospital Adult Primary Care Center with T2D who have *well-controlled* fasting blood sugar levels using basal insulin but who are either not on a GLP-1 RA at all or on a submaximum dose of a GLP-1 RA were enrolled on a secure web platform called Lumeon (Lumeon, Inc). Lumeon sent them an SMS text message every weekday morning at a time they had chosen and that best aligned with their typical morning routine of waking up and checking their blood sugar prior to eating. The SMS text message asked the following: “What was your fasting blood sugar this morning?” Texted responses containing patients’ fasting blood sugar levels were recorded on the Lumeon web platform in a dedicated individual log. On weekdays, the nursing team quickly logged onto Lumeon to check the logs for alarm values. Then, once weekly on Thursdays, the nursing team called patients to review their fasting blood sugar levels, check in regarding any side effects from the GLP-1 RA, consult with the preceptor, and advise patients on whether and how to lower their basal insulin dose and raise their GLP-1 RA dose accordingly. The goals of the program were to decrease insulin, increase GLP-1 RA, and avoid hypoglycemia and hyperglycemia during the transition. The program lasted until the patient had their insulin stopped completely and/or their GLP-1 RA dose reached its maximum, or until 16 weeks elapsed (at which point the patient was discharged from this remote MITI-GLP1 program and returned to usual in-person care with our primary care diabetes team).

Several outcomes were measured. To assess program feasibility and acceptability, patient engagement was evaluated using 2 metrics: the SMS text message response rate and the once weekly call connection rate. The SMS text message response rate was defined as the percentage of SMS text messages sent to patients that received a response. The once weekly call connection rate was defined as the percentage of weeks in which patients were reachable by the MITI-GLP1 nurses for the Thursday titration call. These metrics were chosen because, at a safety-net clinic such as ours, resources are limited, and it is important that new programs are shown to be feasible and acceptable to both patients and the clinic. Preliminary clinical outcomes were also reviewed. In terms of insulin, the percentage of patients who had their insulin dose lowered by at least 50%, as well as the percentage of patients who had their insulin stopped completely, was evaluated. In terms of GLP-1 RAs, the percentage of patients who had their GLP-1 RA dose increased by at least one dosing level, as well as the percentage of patients discharged on the maximum GLP-1 RA dose, was evaluated. The hypoglycemia rate was calculated as the percentage of SMS text message responses with a value of less than 80 mg/dL. Although hypoglycemia is generally defined as any value of less than 70 mg/dL, the MITI-GLP1 program defined hypoglycemia as any value of less than 80 mg/dL out of an abundance of caution [20,21]. Hyperglycemia was defined as any value above 400 mg/dL. As is appropriate for feasibility pilots with small sample sizes, we summarized aggregated metrics rather than using time-series statistical process control, which would examine factors beyond the scope of our pilot.

Key contextual factors were as follows. Throughout the study period, the workload, availability, and competing clinical priorities of MITI nurses were monitored regularly at a weekly team meeting. MITI nurses consulted with preceptors each week in real time before giving titration advice and support to patients over the phone. The overall progress of active patients was reviewed (among MITI nurses, the MITI coordinator, and the program’s medical director) at weekly team meetings.

### Statistical Analysis

Because this quality improvement (QI) review was designed to assess feasibility and acceptability, all analyses were descriptive. Continuous variables are presented as means with SDs, and categorical variables are presented as counts and percentages with 95% CIs. CIs for proportions were calculated using the Wald (normal approximation) method. We did not perform hypothesis testing or make inferential comparisons as the study was not powered for that level of analysis. This approach is consistent with standard reporting practices for pilot and QI work.

### Ethical Considerations

This was a QI review of the MITI-GLP1 pilot program, which was implemented as part of routine clinical care at Bellevue Hospital’s Adult Primary Care Center. Patients were referred by their primary care providers (PCPs) and enrolled through a secure web-based platform called Lumeon, which is supported by NYC Health + Hospitals. Data containing protected health information such as the daily SMS text messages were sent and stored securely by Lumeon. Analyses for the QI review were conducted using deidentified data.

The project was formally classified using the New York University Langone Health Institutional Review Board (IRB) QI self-certification process and met all institutional criteria for QI activities not requiring IRB review. Consistent with definitions in Title 45 of the Code of Federal Regulations §46.102 cited in the institutional policy, the primary objective was to improve local clinical care processes rather than conduct a systematic investigation designed to contribute to generalizable knowledge [22]. The intervention involved no greater-than-routine clinical risk, did not follow a fixed research protocol, was not externally funded as research, and was not part of a multicenter study. In accordance with New York University Langone Health IRB policy, projects meeting these criteria are not considered human subject research and do not require IRB review or oversight.

Given that MITI-GLP1 was a part of routine clinical care, no informed consent was required. There was no compensation for participation.

## Results

The observed contextual factors were as follows. No major policy or staff changes occurred during the pilot period. The American Diabetes Association guidance that a GLP-1 RA–based medication is generally preferred as the first injectable medication in patients with T2D remained the same over the course of the program. No modifications to the process of the intervention were made during the pilot period. The Lumeon texting platform worked reliably for all 72 patients. No technical difficulties interfered with program outcomes.

Data are complete, with all 72 patients represented for each metric.

Table 1 shows MITI-GLP1 patient demographics. A total of 72 patients enrolled in the pilot program. Their average age at baseline was 54 (SD 11) years, with 70.83% (n=51) self-identifying as Hispanic or Latinx, 61.11% (n=44) opting for SMS text messages in Spanish, and 61.11% (n=44) having no health insurance. Of the 72 patients enrolled, 64 (88.89%) completed the program. The remaining 11.11% (n=8) responded to SMS text messages but were unreachable for the titration calls. These 8 patients were discharged from the program and returned to in-person care. All 72 enrolled patients were included in the analyses.

**Table 1.** MITI-GLP1 program patient demographics (N=72).

Demographics	Values
Age (y), mean (SD)	54 (11)
Female sex, n (%)	30 (41.67)
Race, n (%)	
Native American or Alaska Native	0 (0.00)
Asian	0 (0.00)
Black or African American	11 (15.28)
Native Hawaiian or Pacific Islander	1 (1.39)
White	5 (6.94)
Other—Hispanic or Latinx ethnicity	51 (70.83)
Other—Non–Hispanic or Latinx ethnicity	3 (4.17)
No health insurance, n (%)	44 (61.11)
Language used for SMS text messages, n (%)	
Spanish	44 (61.11)
English	25 (34.72)
French	1 (1.39)
Urdu	1 (1.39)
Hungarian	1 (1.39)

Table 2 shows MITI-GLP1 patient engagement outcomes. Patient engagement was meaningfully high; of 3671 SMS text messages sent to patients by the program, 3520 (95.89%; 95% CI 95%‐71%) received a response. Of 719 cumulative weeks during which MITI-GLP1 nurses attempted to reach patients by phone, they were able to connect in 649 (90.26%; 95% CI 88%‐92%).

**Table 2.** MITI-GLP1 patient engagement outcomes (N=72).

Patient engagement outcome	Values
SMS text message response rate	
SMS text messages sent by the MITI-GLP1 program, n	3671
Patient SMS text message responses received, n/N (%; 95% CI)	3520/3671 (95.89; 95‐71)
Once weekly call connection rate	
Weeks with Thursday titration phone calls attempted, n	719
Weeks with Thursday titration phone calls successfully reaching patients, n/N (%; 95% CI)	649/719 (90.26; 88‐92)

Table 3 shows MITI-GLP1 patient baseline clinical information. The average starting basal insulin dose was 20 (SD 11) units once daily, the average starting BMI was 30 (SD 6) kg/m^2^, and the average starting fasting blood glucose level was 126 (SD 19) mg/dL. Among these 72 patients, the GLP-1 RA used was semaglutide in 83.33% (n=60) of patients, dulaglutide in 13.89% (n=10) of patients, and liraglutide in 2.78% (n=2) of patients. Of note, semaglutide was the weekly GLP-1 RA on Bellevue Hospital’s formulary during the pilot period.

**Table 3.** MITI-GLP1 patient baseline clinical information (N=72).

Baseline clinical information	Values
Starting basal insulin dose (units), mean (SD)	20 (11)
Starting BMI (kg/m^2^), mean (SD)	30 (6)
Starting fasting blood glucose (mg/dL), mean (SD)	126 (19)
GLP-1 RA^a^ used, n (%)	
Semaglutide	60 (83.33)
Dulaglutide	10 (13.89)
Liraglutide	2 (2.78)

a GLP-1 RA: glucagon-like peptide-1 receptor agonist.

Table 4 shows MITI-GLP1 preliminary clinical outcomes. Insulin doses were decreased substantially. A total of 76.39% (55/72) of the patients (95% CI 66%‐86%) had their basal insulin dose reduced by 50% or more. In total, 62.5% (45/72) of the patients (95% CI 51%‐74%) had their insulin stopped completely. GLP-1 RA doses increased markedly. A total of 88.89% (64/72) of the patients (95% CI 82%‐96%) had their GLP-1 RA dose increased by 1 level or more, and 62.5% (45/72) of the patients (95% CI 51%‐74%) were discharged on the maximum dose of their GLP-1 RA medication. The average ending fasting blood glucose level was 122 (SD 34) mg/dL.

**Table 4.** MITI-GLP1 preliminary clinical outcomes (N=72).

Outcome	Values
Insulin dose changes, n/N (%; 95% CI)	
Insulin lowered by ≥50%	55/72 (76.39; 66‐86)
Insulin stopped completely	45/72 (62.50; 51‐74)
GLP-1 RA^a^ dose changes	
GLP-1 RA dose increased by at least one level, n/N (%; 95% CI)	64/72 (88.89; 82‐96)
GLP-1 RA dose at discharge was the maximum dose, n/N (%; 95% CI)	45/72 (62.50; 51‐74)
Ending fasting blood glucose (mg/dL), mean (SD)	122 (34)
Hypoglycemia	
SMS text message responses reporting <80 mg/dL, n/N (%; 95% CI)	5/3520 (0.14; 0.02‐0.27)
SMS text message responses reporting <70 mg/dL, n/N (%)	1/3520 (0.03)
Symptomatic hypoglycemia, n/N (%)	0 (0.00)
Hyperglycemia, n/N (%; 95% CI)	
SMS text message responses reporting >400 mg/dL	1/3520 (0.03; 0.00‐0.08)
Symptomatic hyperglycemia	0 (0.00)

a GLP-1 RA: glucagon-like peptide-1 receptor agonist.

Hypoglycemia was minimal. A total of 0.14% (5/3520) of the SMS text message responses (95% CI 0.02%‐0.27%) reported a value of less than 80 mg/dL; 4 of these 5 values were in the range of 70 to 79 mg/dL (79, 79, 74, and 73 mg/dL), and only 1 was below 70 mg/dL (63 mg/dL). None were symptomatic.

Hyperglycemia was also minimal. In total, 0.03% (1/3520) of the SMS text message responses (95% CI 0.00%‐0.08%) reported a value above 400 mg/dL. The value was 494 mg/dL. The patient was asymptomatic.

## Discussion

### Principal Findings

The MITI-GLP1 program is a promising, feasible, and acceptable intervention. Patient engagement was high, as evidenced by both high SMS text message response rates and high weekly call connection rates. Our pilot program sent 3671 SMS text messages to patients asking the following: “What was your fasting blood sugar this morning?” We received a reply 95.89% (3520/3671) of the time. This is notable, especially at a safety-net clinic where social drivers of health often create obstacles to engagement in care. High patient engagement was also reflected in our once weekly call connection rate. MITI-GLP1 nurses attempting to reach patients each week of the program were able to connect with them in 90.26% (649/719) of the weeks. We interpret these metrics to indicate that not only is MITI-GLP1 a convenient and accessible program for *patients* to engage with, it is also a highly feasible program for our *nursing staff*, who were able to successfully reach the patients in their care.

Following American Diabetes Association guidelines, our Adult Primary Care Center generally uses a GLP-1 RA as the first-line injectable agent for most patients with T2D [20,21]. However, the following question arose: what should we do for our patients with T2D who already have *well-controlled* blood sugar levels on basal insulin but who are currently either not on a GLP-1 RA at all or on a submaximum dose of a GLP-1 RA? One option is to simply inform these patients that their blood glucose control is sufficient, refill their medications, and see them back in several months for their next routine visit. Another option is to attempt to transition them to a GLP-1 RA. In our safety-net, primary care internal medicine clinic in New York City, New York, where many of our patients have cardiometabolic and renal disease, we generally prefer the option of attempting to decrease insulin and increase GLP-1 RA dose.

Safely transitioning a person with T2D who has *well-controlled* fasting blood sugar from basal insulin to a GLP-1 RA requires careful monitoring [18,19]. If the basal insulin is not lowered enough by the time the GLP-1 RA starts working, the person may be at risk of hypoglycemia. If the basal insulin is lowered too much before the GLP-1 RA has a meaningful effect, the blood sugar can rise above the goal level of 80-130 mg/dL. In either scenario, the person—who, up until this transition began, had well-controlled fasting blood sugar levels—may lose faith in the new GLP-1 RA medication and may wish to return to their basal insulin, which worked well for them. Our primary care diabetes team began using the MITI-GLP1 program to support this transition, with the overall goals of reducing basal insulin, increasing GLP-1 RA doses, and avoiding both hypoglycemia and hyperglycemia.

Primary care providers do not *need* a MITI-GLP1 program to transition their patients with well-controlled T2D from basal insulin to a GLP-1 RA. Instead, they can advise on dose changes during normal visits and arrange for frequent check-ins (conducted either by themselves or a team member) outside of a formal MITI-GLP1 program. Of note, our primary care diabetes team was doing just that prior to the creation of the MITI-GLP1 program. At any given time, our team knew which patients with *well-controlled* fasting blood sugar were switching from basal insulin to a GLP-1 RA, and these patients remained heavily on our radar during their transition period. We highlighted them at our weekly team meetings and assigned a team member to call them at least once a week to check in about their fasting blood sugar levels and any GLP-1 RA side effects.

For these reasons, including the need to provide multiple titration check-ins and monitor for hypoglycemia and hyperglycemia, we anticipated that a MITI-GLP1 program would successfully support patients and our clinic in the transition from basal insulin to GLP-1 RAs. Indeed, this is what we found.

There are just a handful of publications in the literature describing programs that use SMS text messages to adjust injectable diabetes medications [23-26]. One of those publications reflected the prior work of this team on the original MITI program (which was designed for patients with poorly controlled T2D who needed uptitration of their basal insulin). Beyond the program discussed in our original MITI paper, none of the other studies in the literature were carried out in a safety-net population, none focused on a population who predominantly did not speak the native language, and none involved titration of a GLP-1 RA. What makes MITI-GLP1 most unique is that it is the only study in the literature describing an SMS text messaging–based program whose purpose is to adjust 2 different classes of diabetes medications simultaneously: basal insulin and GLP-1 RAs.

The implications of our work are several-fold. First, receiving daily SMS text messages allows a team to keep a close watch for hypoglycemia and hyperglycemia. There were only 5 cases of hypoglycemia; there was only 1 case of hyperglycemia. However, our team knew about these abnormal glucose values right away and could contact patients and make medication adjustments in real time.

Second, having the fasting blood glucose data gathered in advance changes the quality of the weekly telephone interaction by allowing the team to focus on education and titration rather than on data gathering. Health care providers tasked with calling patients to check in about self-monitored blood glucose values and advise on medication adjustments know that check-in calls often require a significant amount of time to elicit the recent blood glucose values. Patients may not be home when called and, thus, may have to recreate a log of recent blood sugar levels from memory. Alternatively, patients may be home, but it still takes ample time to scroll back through the values on their glucometer or continuous glucose monitor. In contrast, having accurate fasting blood glucose values in front of the health care provider at the start of the phone call makes the call more efficient and allows the health care provider to devote more time to check in about any GLP-1 RA side effects, give medication titration advice, and offer general support.

Finally, having a program such as MITI-GLP1 can be a factor that encourages PCPs to recommend the labor-intensive transition in the first place, especially given time constraints in a safety-net clinic. Without a MITI-GLP1 program, a PCP may evaluate their patient with well-controlled T2D who is on basal insulin *but not on a GLP-1 RA* and may recognize, given their patient’s high cardiometabolic and renal risk, that it would be *better* for their long-term health to attempt the transition to a GLP-1 RA. However, in the context of a 20-minute visit, it can be challenging for the PCP to find the time to put that plan into motion. Thus, the decision to do so may be deferred. With the existence of the MITI-GLP1 program, PCPs can devote *more of their time* to obtaining patient buy-in by educating them about the reasons why a GLP-1 RA may be superior to basal insulin and *less of their time* on titration logistics. Specifically, the presence of the MITI-GLP1 program can eliminate the need for the PCP to arrange a series of follow-up appointments to check in about potential GLP-1 RA–related gastrointestinal symptoms and provide ongoing titration advice. If the patient agrees to switch over from insulin to a GLP-1 RA, the PCP can simply refer the patient to our program. Conveniently, we take it from there.

The MITI-GLP1 program systematically operationalizes the transition process, taking it off the shoulders of the *individual* PCP and assigning it instead to a multidisciplinary *team* that is not only well versed in transitioning patients from basal insulin to a GLP-1 RA but also able to use convenient, patient-centered SMS text messaging and a once weekly phone call to provide much more continuous guidance, support, and supervision during the process.

This work is the first description in the literature of an SMS text messaging–based program that aims to titrate both basal insulin and GLP-1 RAs. The MITI-GLP1 program was feasible and acceptable, demonstrated strong preliminary outcomes, and warrants further investigation in a larger clinical trial.

Program costs include the cost of the secure SMS text messaging platform (Lumeon) and the cost of the program coordinator. While there is a “cost” to the nursing time as well as the time of the preceptor involved in titration discussions, the authors argue that these are not opportunity costs because they would be incurred whether a patient was having these medications adjusted via the MITI-GLP1 program or outside a formal MITI-GLP1 program.

### Limitations

The MITI-GLP1 pilot program has several limitations. First, the program’s small sample size limits generalizability and power. Second, the lack of a control group prevents attribution of the stability of blood glucose values to the intervention. Third, the short follow-up period provided in the MITI-GLP1 program is insufficient to assess long-term durability of glycemic control. Additionally, the scalability of the intervention may be impacted by staff differences across clinics. This program was carried out by diabetes-focused primary care nurses who are considerably experienced and comfortable in managing injectable medications for people with complex T2D. Not all primary care nurses will share this comfort level. Finally, a MITI-GLP1 program requires access to a secure SMS text messaging platform. We are lucky to have access to Lumeon in our health care system and realize that not everyone will. Of note, it was important that SMS text messages sent by our program through Lumeon were standard SMS text messages only requiring the patient to have a cell phone, a regular SMS text messaging plan, and a basic understanding of how to receive and send SMS text messages. We strongly preferred this to a platform requiring patients to own a smartphone, download an app, or have access to Wi-Fi as these would have been additional barriers for our safety-net patients.

### Conclusions

“Well-controlled T2D” in 2026 does not merely refer to healthy blood glucose values, but rather, it refers to effective glycemic control in the broader context of medications that reduce cardiometabolic and renal complications. A primary care internal medicine–run MITI-GLP1 program using SMS text messaging and teamwork between internists and nurses is a feasible and acceptable intervention for safely and effectively transitioning people with well-controlled T2D away from basal insulin and toward a GLP-1 RA. Further confirmatory studies, such as those involving a larger controlled trial, are needed.

## Abbreviations

GLP-1 RA: glucagon-like peptide-1 receptor agonist
IRB: institutional review board
MITI: Mobile Insulin Titration Intervention
PCP: primary care provider
QI: quality improvement
T2D: type 2 diabetes
